# Usability and Impact of the Web-Based Dementia Foundations Educational Program in Personal Support Workers (PSWs), PSW Trainees, and Care Companions: Quasi-Experimental Study

**DOI:** 10.2196/67889

**Published:** 2025-07-21

**Authors:** Anthony J Levinson, Stephanie Ayers, Sandra Clark, Patricia Gerantonis, Amy Schneeberg, Richard Sztramko

**Affiliations:** 1 Division of e-Learning Innovation, Faculty of Health Sciences, McMaster University, 1280 Main Street West, Hamilton, ON, L8S 4L8, Canada, 1 (905) 525-9140 ext 26094; 2 Freelance Statistical Consultant, Vancouver, BC, Canada; 3 Department of Medicine, McMaster University, Hamilton, ON, Canada

**Keywords:** dementia, caregivers, e-learning, education, training, mobile phone

## Abstract

**Background:**

Personal support workers (PSWs) are often expected to provide ongoing support for complex conditions and have identified an increased need for training in several areas, including dementia and mental health. Web-based interventions may be helpful complements to traditional in-person continuing education and training, but their effectiveness must be explored further.

**Objective:**

This study’s objective was to evaluate the usability, usefulness, satisfaction with, and effectiveness of the web-based Dementia Foundations Program among unregulated care providers who provide care to persons living with dementia or are in training.

**Methods:**

A cohort of 50 PSWs, PSW trainees, and paid care companions from 3 recruitment sites were invited to access the Dementia Foundations Program, a 4-hour self-paced web-based program composed of 4 courses, for up to 6 weeks. Usability, usefulness, and satisfaction were assessed using surveys after each course and following the program. Dementia knowledge and attitudes were measured using the Dementia Knowledge Assessment Scale and the Dementia Attitudes Scale, with differences between baseline and postprogram scores analyzed using repeated measures ANOVA.

**Results:**

Participants reported high levels of satisfaction with the program. Of the 50 participants, 46 (92%) agreed that the web-based training met their expectations, 47 (94%) agreed that the training covered a broad range of topics and was not missing any important content, and 49 (98%) agreed that the web-based training would benefit them. There was a significant postprogram improvement in dementia knowledge as measured by the Dementia Knowledge Assessment Scale, with an average 30% improvement across all cohorts. Dementia Attitudes Scale scores were also significantly improved postprogram across all cohorts.

**Conclusions:**

This pilot study in PSWs, PSW trainees, and unregulated care companions demonstrated high satisfaction levels with the web-based Dementia Foundations Program. There were substantial improvements in knowledge and small improvements in attitudes for participants, and it was perceived as a useful tool that complemented their existing education and training. The Dementia Foundations Program is a user-friendly and effective e-learning program, which can be conveniently scaled and spread to enhance unregulated care provider dementia education.

## Introduction

### Background

The number of older adults aged 60+ years is expected to double worldwide by 2050 [1]. In Canada specifically, the older seniors population aged 75+ years is expected to grow even sooner, possibly tripling by 2046 [2]. This has the potential to increase the prevalence of age-related health conditions such as dementia, as well as the need for adequate human health resources [3].

### The Need for Unregulated Care Providers

There are estimated to be about 600,000 people in Canada living with dementia currently, with that number projected to grow to almost a million in 2030 [4]. Unregulated care professionals who provide care to older adults play an important role in the effort to meet the growing demand for care from Canada’s aging population. Several occupational titles can be used to describe these unregulated care providers, including personal support workers (PSWs), health care aides, and home care workers [5]. For the purposes of this paper, we will predominantly use the term PSWs, with the intent that this information applies to most professionals working in similar unregulated care roles.

It is estimated that there are over 1 million PSWs in Canada [6] providing support, including assisting with daily living activities, light housekeeping, meal preparation, socialization, and companionship [7], in several settings, including home, community, and clinical. In Canada, PSWs are not currently required to hold accredited qualifications and are not formally regulated by an organization or governing body. This lack of regulation of training can lead to gaps in knowledge and caregiving abilities [6]. PSWs have indicated that more training is needed, specifically in the field of dementia and mental health [8]. With over 69% of residents in long-term care (LTC) homes living with dementia and 87% with some form of cognitive impairment [9], more specific education and training about dementia are needed for PSWs.

### Dementia Education Approaches

There are currently a variety of training approaches for dementia for unregulated care providers. Some PSW training programs or colleges might offer traditional didactic instruction on topics related to dementia; some of these programs have transitioned to webinar-based instruction during the pandemic. For PSWs or other unregulated care providers already in the workplace, the most common approach would be occasional on-the-job training, such as periodic “in-service” or “lunch-and-learn” sessions. These approaches tend to be synchronous, nonstandardized, and not necessarily educationally designed. Very few offer foundational knowledge content about dementia. Some approaches may also involve face-to-face instruction, which did prove to be challenging during the pandemic, concerning public health guidance as well as participant hesitance, even with the loosening of public health guidance [10]. Many of those solutions would be both challenging to scale and likely inconvenient for these care providers, who may not have flexible schedules that can accommodate synchronous instruction, especially if expected to be done on unpaid (personal) time.

For these reasons, the Dementia Foundations Program was developed in 2021 at McMaster University, aligning with several performance objectives and standards of the PSW Program Standard for Ontario Colleges from the Ministry of Training, Colleges and Universities [11]. During program development, project team members and target audience stakeholders identified the need for flexible, asynchronous solutions to best facilitate delivery to their PSW employees and students.

### Program Description

The Dementia Foundations Program is a web-based, asynchronous (self-paced) series of 4 courses that takes approximately 4 hours total to complete. Each of the 4 courses includes modules on various topics related to dementia and dementia care and is described in Table 1.

**Table 1.** Dementia foundations program course descriptions.

Course number and course name	Course content
1. Foundations of Dementia	• What cognition is and how it is impacted by dementia • Difference between normal aging and mild cognitive impairment • Different types and stages of dementia • Types of treatments available
2. Responsive Behaviors and Mental Health	• Psychiatric issues such as apathy, depression, and anxiety that may affect people with dementia • Behavioral, emotional, and psychiatric symptoms of dementia • Strategies to help manage responsive behaviors
3. Home Supports and Safety	• Identifying safety risks • Strategies to reduce harm • Home and community-based supports and services available for patients and families
4. Promoting Brain Health and Caregiver Wellness	• Importance of brain health • How to make positive brain health choices • Why caregiver wellness is important and what can be done to support it

The program is intended as a complementary dementia education resource that may benefit care providers’ training for several reasons. First, the modules within the courses use high-quality, evidence-based, multimedia instructional design [12,13], which has been shown to improve learning outcomes. Second, it can be delivered asynchronously for self-paced learning at any time and from any place, unlike other training programs that may use real-time training formats such as instructor-led classroom or webinar training. Third, the program has been developed using responsive web design and is compatible with any internet-connected device (smartphone, tablet, or desktop). Fourth, it is more efficient and cost-effective to scale than standardized digital or in-person training, as it does not require the presence of instructors. Lastly, the Dementia Foundations Program offers a certificate of completion for each course and the complete program that learners can share with future employers detailing the time they have invested in learning about caring for those living with dementia.

### Study Purpose

The objective of this study was to evaluate the usability, usefulness, satisfaction, and effectiveness of the Dementia Foundations Program among unregulated care providers who currently provide care to, or could provide care to, persons living with dementia.

## Methods

### Study Design

A pilot pre-post design was used with measures taken at baseline, after each of the 4 courses, and after program completion. Participants were able to complete the program at their own pace over a maximum of 6 weeks.

### Participants and Recruitment Cohorts

A total of 50 participants were recruited through collaborative partner networks via an email campaign, keeping in line with typical pilot study sample size guidance in the literature [14,15]. Participants were organized into 3 recruitment cohorts: those working in the city of Ottawa’s LTC homes, those currently a part of Durham College’s PSW training program, and those recruited from the uCarenet homecare digital marketplace. Participants who fulfilled the following inclusion criteria were eligible to participate: (1) had a good command of the English language, (2) were sufficiently computer literate to use the Dementia Foundations Program, and (3) were currently an unregulated health care provider or trainee in Ontario, Canada. Interested participants were directed to a private web-based sign-up link where they were provided further study information and could submit informed consent. Once informed consent was received, a member of the research team manually added participants to the Dementia Foundations Program within the learning management system. Participants were grouped according to their recruitment cohort to allow the research team to identify and analyze patterns and trends among these 3 different settings.

### The Dementia Foundations Program

Participants had access to the Dementia Foundations Program from June 1 to July 9, 2021. The program could be accessed from any internet-enabled device at any time or location. In addition to the 4 courses, participants in this pilot study had access to a “Getting to Know You” course at the beginning (which included the baseline assessments), and a “Review and Final Quiz” course at the end, which included the postprogram assessments.

### Program Usability, Usefulness, and Satisfaction

To evaluate perceived usability, usefulness, and satisfaction with the training, 2 questionnaires were developed based on the Information Assessment Method for all questionnaire—a content-validated questionnaire designed to collect feedback from health information consumers based on 4 domains: situational relevance, cognitive impact, information use, and health benefits [16]. The first was administered after each course (postcourse assessment), and the second was delivered upon program completion (postprogram assessment). All questions were required, and each survey could only be submitted once; participants were able to review their responses and were asked to complete any questions they had missed before submitting.

The postcourse assessment consisted of 7 questions in total: 4 questions that could be answered on a 5-point Likert scale ranging from “strongly agree” to “strongly disagree” (“I thought the course content was very important to my professional/learning needs” [usefulness], “I understood the content in this course” [usability], “I was able to complete the course in a reasonable amount of time” [usability], and ”I would recommend this course to a colleague” [usefulness and satisfaction]), 2 questions that could be answered with multiple-select answers (”What do you think about this course?” [usefulness] and “Which benefit(s) are you expecting after taking this course?” [usefulness]), and 1 open text question (“Any other comments about this course?” [usability, usefulness, or satisfaction]).

The postprogram survey consisted of 8 questions total: 4 questions that could be answered on a 5-point Likert scale ranging from “strongly agree” to “strongly disagree” (“This online training met my expectations” [satisfaction], “I felt that this training covered a broad range of topics and was not missing any important content or topics” [usefulness and satisfaction], “This online training will benefit me” [usefulness and satisfaction], and “I feel my certificate/credential related to this program will be very valuable to me with current or future employers” [usefulness and satisfaction], 1 question that could be answered with a 3-point Likert scale ranging from “too low” to “too high” (thoughts on proposed pricing structure), 1 question that could be answered with multiple-select options (“Which of the following courses would you consider paying for?”), and 2 open text questions (“How much would you be willing to pay for this program?”, and “Is there anything else you would like to say about this program?” (usability, usefulness, or satisfaction).

### Dementia Knowledge

To evaluate the effectiveness of the Dementia Foundations Program, change in knowledge and attitudes related to dementia was assessed. Knowledge was measured at baseline and after program completion with the Dementia Knowledge Assessment Scale (DKAS; reliability *α*=.85; ω_h_=0.87; overall scale) [17]. The DKAS consists of 25 items on different aspects of dementia that could be answered with “true,” “probably true,” “false,” “probably false,” or “I don’t know.” The total scores achievable for this scale range from 0 to 50, with greater dementia-related knowledge reflected by a higher score.

### Dementia Attitudes

Attitudes about dementia were measured at baseline and after program completion with the Dementia Attitudes Scale (DAS) [18]. The DAS consists of 20 items on a 7-point Likert scale that reflect the affective, behavioral, and cognitive components of the attitudes toward individuals with Alzheimer disease and related dementias. The total scores achievable for this scale range from 20 to 140, with a more positive attitude reflected by a higher score.

### Learning Management System Setup

All assessments were offered digitally within a learning management system. The baseline “Getting to Know You” course included a demographic questionnaire (age, sex, race or ethnicity, highest level of education completed, occupation, and most common workplace setting), the DKAS, and the DAS. The postcourse assessment was built directly into the end of each of the 4 courses. The postprogram “Review and Final Quiz” course included the DKAS, DAS, and postprogram assessment.

### Data Analysis

Descriptive analyses were used to summarize the baseline characteristics of the study population. Differences between baseline and postintervention DKAS and DAS scores for all groups were analyzed using repeated measures ANOVA. All analyses were performed using R (version 4.4.1; R Foundation; June 14, 2024).

### Ethical Considerations

The Hamilton Integrated Research Ethics Board reviewed the study protocol and granted exemption from full review on March 18, 2021, as this was considered a quality improvement initiative. Participants were required to provide informed consent. All participants were informed of the length of time of the e-learning and surveys, as well as details surrounding data collection, storage, and investigator identities. Participants’ identities and confidentiality were maintained throughout the research study. All participant data were deidentified and were stored on password-protected secure servers to prevent unauthorized access. There was no known risk or harm to participating in this study or publicizing its results or findings. Upon program completion, participants were provided with a CAD $80 (US $58.22) gift card and a certificate of completion for their professional portfolio.

## Results

### Participant Characteristics

A total of 50 unregulated care providers enrolled and completed this study. Of these, 24 (48%) participants identified as PSWs, 16 (32%) identified as a PSW trainee or student, 1 (2%) identified as a health care aide, 1 (2%) identified as a personal care assistant, 4 (8%) identified as other front line health care workers, 1 (2%) identified as unregulated care provider manager, and 3 (6%) identified as other (while with no further details on what their role was, care settings identified were retirement home or LTC [recruited from the Durham College cohort], group home [recruited from the uCarenet cohort], and possibly for a family member [recruited from the uCarenet cohort] for these 3 participants). Participant demographics broken down by the 3 recruitment cohorts are outlined in Table 2.

**Table 2.** Participant demographics in each recruitment cohort.

Characteristics	Number of responses per recruitment cohort		
	City of Ottawa LTC^a^ (n=20), n (%)	Durham College (n=17), n (%)	uCarenet (n=13), n (%)
Age (years)			
18 to 24	1 (5)	2 (12)	0 (0)
25 to 34	2 (10)	9 (53)	5 (38)
35 to 44	9 (45)	3 (18)	4 (31)
45 to 54	5 (25)	3 (18)	2 (15)
55 to 64	3 (15)	0 (0)	2 (15)
Highest level of education completed			
High school	0 (0)	2 (12)	0 (0)
College or university	17 (85)	15 (88)	13 (100)
Graduate school	3 (15)	0 (0)	0 (0)
Role			
PSW^b^	19 (95)	0 (0)	5 (38)
PSW trainee or student	0 (0)	16 (94)	0 (0)
Health care aide	1 (5)	0 (0)	0 (0)
Personal care assistant	0 (0)	0 (0)	1 (8)
Other front-line health care worker	0 (0)	0 (0)	4 (31)
Unregulated care provider manager or administrator	0 (0)	0 (0)	1 (8)
Other	0 (0)	1 (6)	2 (15)

a LTC: long-term care.

b PSW: personal support worker.

### Program Usability, Usefulness, and Satisfaction

Overall, the courses were positively rated, with most participants agreeing that the course was “very important” to their professional and learning needs (Table 3). Participants agreed that the training benefited them by “improving the health and well-being of the people that they care for.” Additionally, participants identified that the training allowed them to “feel more confident,” “prevent a problem,” “handle a problem or the worsening of a problem,” and “decide something with someone else.” The participants were also asked to provide any additional comments about the course; many open-text responses were very positive. Participants identified the importance and applicability of the information to their professional work.

In general, participants reported high levels of satisfaction with the program. A total of 46 of 50 (92%) of participants agreed or strongly agreed that the web-based training met their expectations, 47 (94%) agreed or strongly agreed that the training covered a broad range of topics and was not missing any important content, 49 (98%) agreed or strongly agreed that the web-based training would benefit them, and 46 (92%) agreed or strongly agreed that the program would be very valuable with current or future employers. No support inquiries were received from participants, further confirming the high usability of the program.

**Table 3.** Summary of postcourse feedback.

Question and course		Response breakdown, n (%)				
		Strongly disagree	Disagree	Neither agree nor disagree	Agree	Strongly agree
I thought the course content was very important to my profession/learning needs						
	Course 1 (Foundations of Dementia)	4 (8)	0 (5)	1 (2)	18 (36)	27 (54)
	Course 2 (Responsive Behaviours and Mental Health)	0 (0)	0 (0)	0 (0)	20 (40)	30 (60)
	Course 3 (Home Supports and Safety)	1 (2)	1 (2)	1 (2)	19 (38)	28 (56)
	Course 4 (Promoting Brain Health and Caregiver Wellness)	0 (0)	1 (2)	1 (2)	28 (56)	20 (40)
I was able to complete the course in a reasonable amount of time.						
	Course 1 (Foundations of Dementia)	0 (0)	0 (0)	7 (14)	24 (48)	19 (38)
	Course 2 (Responsive Behaviours and Mental Health)	0 (0)	0 (0)	2 (4)	29 (58)	19 (38)
	Course 3 (Home Supports and Safety)	0 (0)	1 (2)	5 (10)	22 (44)	22 (44)
	Course 4 (Promoting Brain Health and Caregiver Wellness)	1 (2)	0 (0)	3 (6)	28 (56)	18 (36)
I would recommend this course to a colleague.						
	Course 1 (Foundations of Dementia)	0 (0)	0 (0)	2 (4)	13 (25)	35 (70)
	Course 2 (Responsive Behaviours and Mental Health)	1 (2)	0 (0)	2 (4)	19 (38)	28 (56)
	Course 3 (Home Supports and Safety)	0 (0)	0 (0)	0 (0)	19 (38)	31 (62)
	Course 4 (Promoting Brain Health and Caregiver Wellness)	0 (0)	0 (0)	1 (2)	20 (40)	29 (58)

### Change in Dementia Knowledge

**Figure 1.**
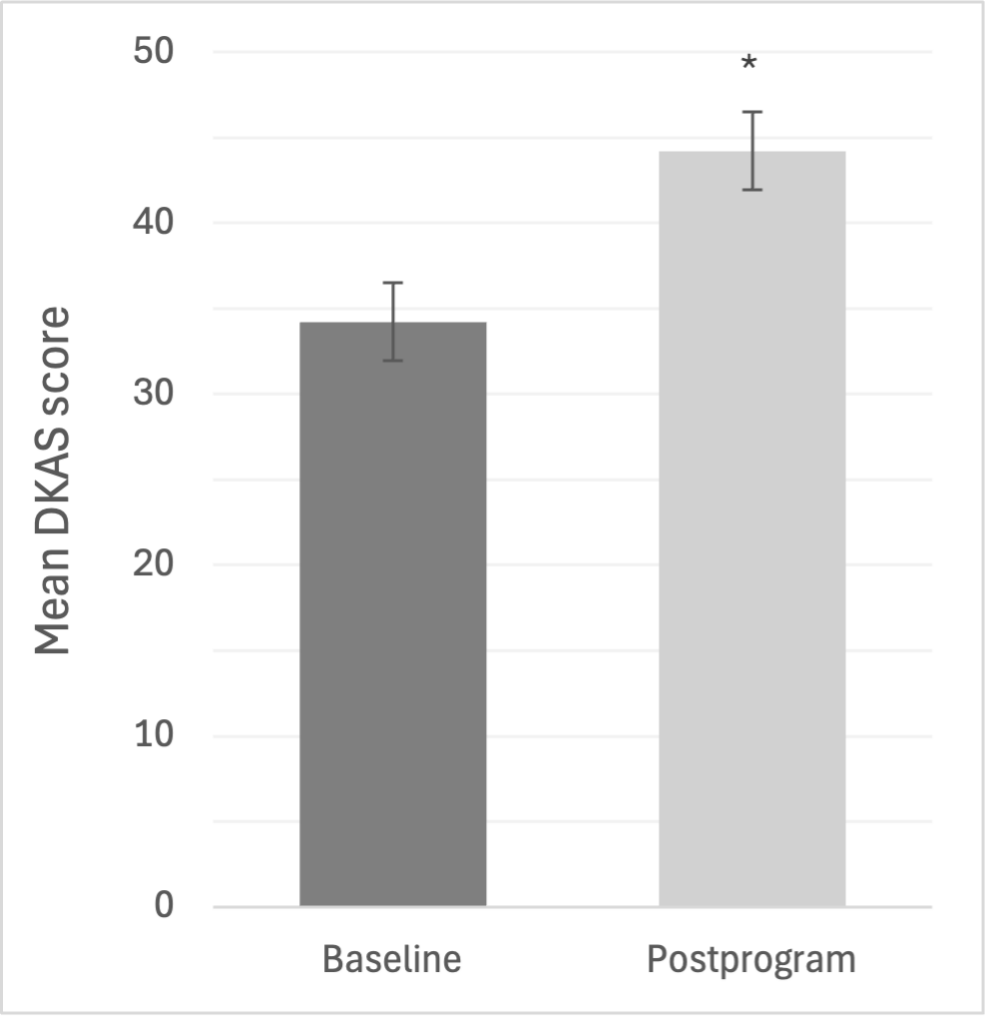
Baseline and postprogram DKAS scores for all participants. Results of repeated measures ANOVA (mean with 95% CI) with time as the only predictor of DKAS, including a random effect for participant ID. *Represents statistical significance (*P*<.001). DKAS: Dementia Knowledge Assessment Scale.

**Figure 2.**
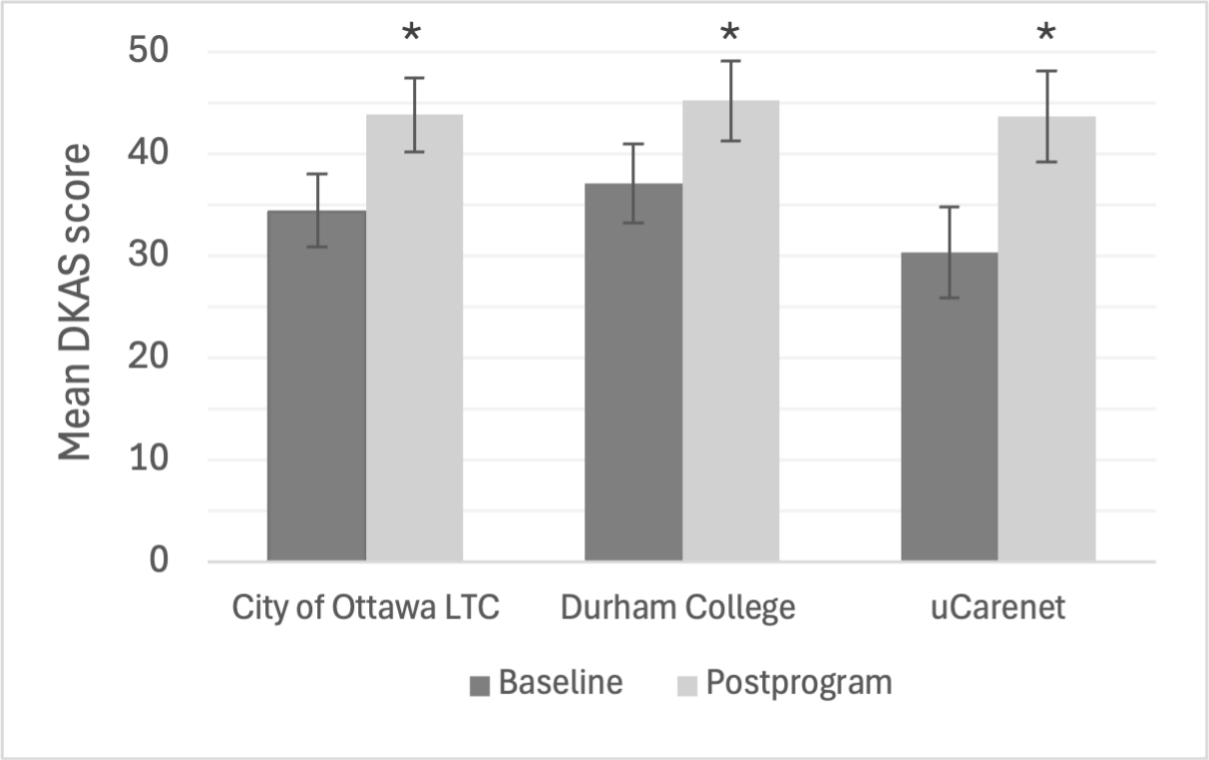
Baseline and postprogram DKAS by cohort and time. Results of repeated measures DKAS model (mean with 95% CI) with cohort time interaction, including a random effect for participant ID. *Represents statistical significance (*P*<.05). DKAS: Dementia Knowledge Assessment Scale; LTC: long-term care.

All 50 participants completed the baseline and postprogram DKAS. One participant from the City of Ottawa LTC recruitment cohort was missing data for 15/25 items on the baseline DKAS due to a technical glitch. The most conservative approach was taken where it was assumed the participant would have received 2 points for each missing answer (ie, the highest score possible). The average baseline DKAS score was 34.2 of 50 (68%, SD 10.5) and the average postprogram score was 44.2 of 50 (88.4%, SD 4.7). Scores for all participants are shown in Figure 1, with scores for each recruitment cohort shown in Figure 2.

Repeated measures ANOVA showed a statistically significant effect on DKAS scores (*F*_1,49_=58.533, *P*<.001), with scores increasing from baseline to postprogram across all 3 recruitment cohorts ($\eta^{2}=$0.276). The model, including only time as a predictor and a random effect for participant ID, demonstrated that relative to baseline, there was an average 9.98-point increase on the DKAS score postintervention (95% CI 7.88-12.08; SE 1.065; *P*<.001). Post hoc testing did not reveal any significant differences in DKAS scores between recruitment cohorts; the model including an interaction with time and cohort, and a model with just time (not including cohort at all), were not statistically significantly different (*P*=.24).

### Change in Dementia Attitudes

**Figure 3.**
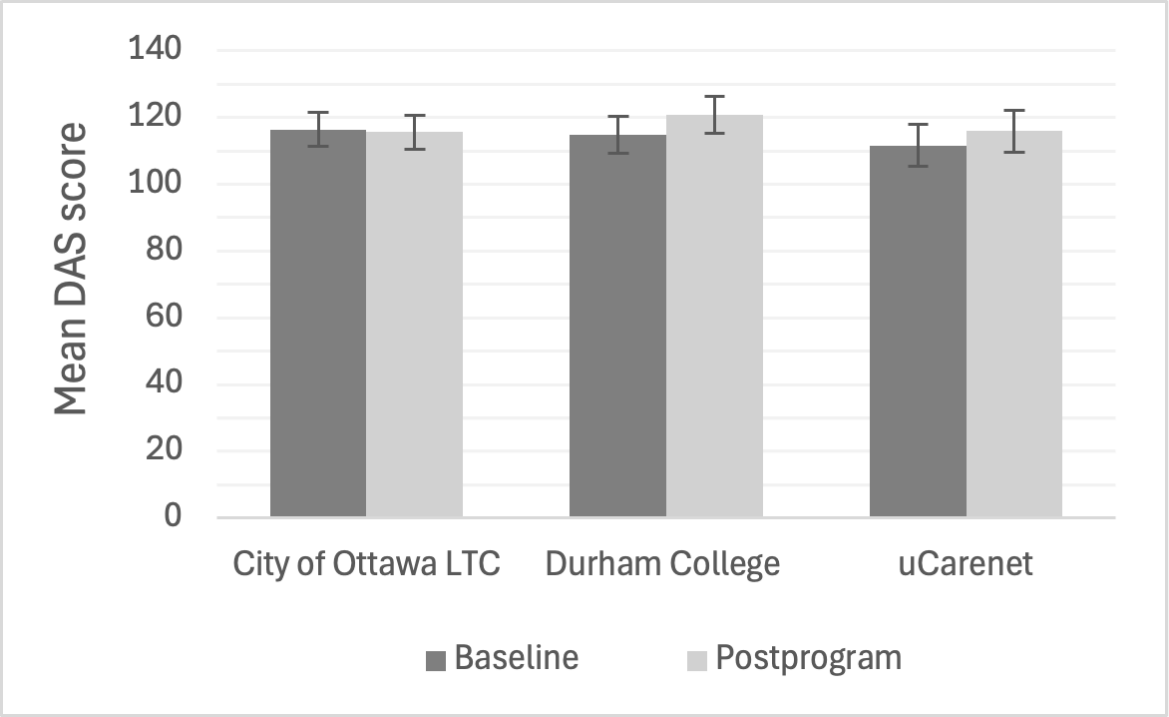
Baseline and postprogram DAS scores among participants by cohort. Results from repeated measures DAS model (mean and 95% CI) including interaction for cohort and time. DAS: Dementia Attitudes Scale; LTC: long-term care.

All 50 participants completed the baseline and postprogram DAS. On average across all recruitment cohorts, participants scored 114.7 of 140 (82%; SD 11.6) at baseline and 117.5 of 140 (84%; SD 11.2) postprogram. Scores for each recruitment cohort are shown in Figure 3.

Repeated measures ANOVA showed a statistically significant effect on DAS scores (*F*_1,49_=4.383, *P*=.04; $\eta^{2}=$ 0.015). The model including only time as a predictor demonstrated that relative to baseline, there was an average 2.8 point increase on the DAS score postprogram (*P*=.04). Post hoc testing did not reveal any significant differences in DAS scores between recruitment cohorts; the model including an interaction with time and cohort, and a model with just time (not including cohort at all) were not statistically significantly different (*P*=.15).

## Discussion

### Principal Findings

The purpose of this pilot study was to evaluate the usability, usefulness, satisfaction, and effectiveness of the Dementia Foundations Program, a series of 4 web-based courses about various aspects of dementia developed for unregulated care providers such as PSWs. Three sites were recruited from slightly different populations (PSW trainees, PSWs working in LTC, and PSWs and other unregulated care providers working in community settings) who were all willing to take part in recruitment efforts due to the perceived benefit of additional dementia care training for their students, employees, and members. Overall, the program was positively valued for its usability, and participants reported a high level of satisfaction with the program. Participant knowledge, as measured by the DKAS, significantly increased after completing the program, with an average 30% improvement across all recruitment cohorts. A statistically significant increase was also seen for dementia attitudes as measured by the DAS, with a more modest average improvement of 2.5%. While scores for both the DKAS and DAS did not significantly differ between cohorts, there are a few trends worth highlighting.

PSW trainees from the Durham College cohort had higher baseline DKAS scores and slightly lower DAS scores compared to the PSWs from both the City of Ottawa LTC and uCarenet cohorts. The differences in knowledge scores could be explained by how trainees might have higher fact-based knowledge (from more recent studying) and may be more used to taking knowledge assessments, whereas practicing PSWs may have more everyday clinical knowledge and may not have taken a formal course in some time. When looking at dementia attitudes, the overall significant increase can be attributed to the Durham College and uCarenet cohorts, rather than the Ottawa LTC cohort. This conceptually makes sense, as practicing PSWs may already possess more developed attitudes toward dementia that would not be expected to change drastically after only 4 hours of knowledge-based education. Conversely, PSW trainees and PSWs not working in LTC homes who have had less exposure to working with people with dementia may be more prone to shifting their attitude in a significant way after 4 hours of education.

### Comparison to Prior Work

#### Dementia Education

There is currently a lack of dementia-specific care training offered to unregulated care providers, leading to gaps in care [8,19]. This highlights the need for increased and enhanced training to improve education with the purpose of improving care for individuals with dementia. Research has shown that dementia education and training for health care workers improves knowledge, fosters positive attitudes, increases confidence, and ultimately produces better outcomes for individuals living with dementia [20,21]. An extensive review by Surr et al [22] evaluated various features of dementia education programs for health care providers. Among the findings, the authors concluded that effective dementia education programs should be relevant to the workers’ role and experience, include practice-based learning with theoretical content, be delivered by an experienced facilitator, and involve in-person facilitation. Although there are some existing on-the-job dementia training programs for unregulated care providers, they vary in availability, are more expensive to deliver, and are often less convenient for care providers who may not have flexible schedules to accommodate this type of instruction. The COVID-19 pandemic also imposed limitations on access to many in-person education programs. Thus, a blended knowledge approach that combines in-person training with web-based learning can deliver feasible and effective dementia education with the potential to be scaled and spread to a large population.

Furthermore, the recommendation by Surr et al [22] for in-person delivery may be influenced by several factors. For example, it is likely the case that most education and training programs about dementia have historically been delivered in person. Second, those programs delivered digitally (whether synchronously through webinar platforms or asynchronously) may not have incorporated best practices concerning the instructional design of multimedia e-learning. High-quality instructional design has been shown to improve learning transfer. The Dementia Foundations Program was created using evidence-based principles for multimedia learning outlined by Mayer [12] and Clark and Mayer [13]. The findings from the current study indicate that this entirely asynchronous web-based program is a useful and effective training modality for improving dementia-related knowledge among unregulated care providers. The high satisfaction with the program is evident in the course open-text comments, where most participants praised the program for its informativeness, user-friendliness, and usefulness to their profession as unregulated care providers.

#### About DKAS

Significant improvements in dementia knowledge were seen after the intervention for both PSWs and PSW trainees. As unregulated care providers in Canada have noted a lack of dementia-specific care training and education, this indicates that the Dementia Foundations Program is an effective way to impact dementia knowledge for this group. Other web-based dementia education programs have also been shown to be effective in increasing dementia knowledge. Eccleston et al [23] evaluated the efficacy of the 9-week-long Understanding Dementia Massive Open Online Course (UDMOOC) for people with varying professional and educational backgrounds. Similar to the Dementia Foundations Program, the UDMOOC aims to improve dementia knowledge by teaching a broad community audience basic neurobiology, dementia pathophysiology, medical management, and person-centered care. The median DKAS score at baseline for their study was 34.5 out of 50, which is comparable to our current pilot study’s baseline median DKAS score of 35.5 out of 50, implying both groups started with similar levels of dementia knowledge before embarking on the training. The median postprogram scores for both studies were 45 of 50, both demonstrating a significant increase in dementia knowledge. These results are consistent with the current study’s findings, indicating that web-based dementia education modalities are effective at increasing dementia knowledge. However, it is worth noting that the Dementia Foundations Program can be completed in a single day with only 4 hours’ worth of content, whereas the UDMOOC estimates 21 hours over a 7- to 9-week period. Comparable baseline and postprogram median DKAS scores for both programs suggest the Dementia Foundations Program is more efficient for busy professionals, with similar knowledge gain in a much shorter amount of time.

When considering the mode of delivery of instruction, 1 study conducted by Parveen et al [24] also found that the most impactful dementia training programs involved a blend of both e-learning and in-person delivery, although another study conducted by Vollmar et al [25] found no significant differences between learning through in-person compared with web-based learning. With the significant increase in dementia knowledge shown by both the current study and Eccleston et al using only a web-based approach, it is reasonable to consider digital-only approaches to some aspects of dementia education, especially when considering the increased flexibility this offers to an unregulated care worker audience with potentially variable and less flexible schedules. A blended approach has been shown to be effective in other studies, and one can speculate that an efficient and effective approach might involve high-quality asynchronous web-based approaches for baseline knowledge education, complemented by in-person skills training.

#### About DAS

Dementia attitudes significantly increased from baseline to postprogram, with scores moving from an average of 114.7 to 117.5. While this increase is modest at 2.5%, baseline scores on the DAS started quite high compared to other studies looking at dementia attitudes in nursing students in Malta (mean DAS score of 107.9) [26] and health care professionals in China (mean DAS score of 91.3) [27]. The highest possible score on the DAS is 140, corresponding to the highest possible positive attitude toward dementia. The DAS scores from the current study indicate that this sample of participants already possesses relatively positive attitudes toward dementia compared to previous studies, therefore leaving only modest room for improvement on this measure.

Baseline DAS scores averaged 114.7 across all 3 recruitment cohorts, with uCarenet starting with the lowest score of 111.6 and the City of Ottawa LTC starting with the highest score at 116.4. After the 4-hour intervention, DAS scores were significantly higher at 117.5 across all 3 recruitment cohorts, with the Durham College PSW trainees showing the largest increase among cohorts from 114.9 to 120.9. Logically, this cohort showed the biggest increase in positive attitudes toward dementia, as trainees have very likely spent less time working with older adults and those with dementia. The experienced PSWs and other paid caregivers that comprised the Ottawa LTC and uCarenet cohorts have likely been working with people with dementia for many years and have had ample time to develop personal attitudes toward dementia, as previous research has shown that working with and having ongoing and meaningful interactions with individuals living with dementia are key to fostering a change in attitudes [26,28]. It can therefore be reasonably assumed that 4 hours’ worth of independent learning is not a sufficient intervention length to cause an attitude change in experienced workers, but remains sufficient exposure to improve attitudes toward dementia among trainees. This contrasts with a study that explored the impact of a single dementia awareness session on changing dementia attitudes among adolescents, with no significant improvements in either the intervention or control groups [29]. However, the difference may be that the Durham College PSW trainees were more accepting of a potential attitude change toward dementia given their desired career choice as a PSW.

### Limitations

We acknowledge that there are limitations to this study. First, the relatively small sample size of 50 PSWs, PSW trainees, and other unregulated care professionals from Ontario who took part in this study is not necessarily representative of the diverse and large population of unregulated care providers in Canada or internationally. However, as the sample size was based on the typical large effect sizes for e-learning interventions, the results of this study remain valuable for an initial assessment of a new e-learning program for this population. This was further confirmed by the detection of statistically significant changes in both dementia knowledge and attitudes as measured by the DKAS and DAS. Second, the lack of a control group due to the pre-post study design introduces an unknown risk of bias; however, the pre-post design does alleviate intraparticipant variability and is consistent with the majority of studies in a 2019 systematic review of studies assessing technology-delivered dementia education to health care providers [30]. Lastly, not all participants had active experience working with people living with dementia, and as a result, may not have found the learning content to be relevant. However, trainees were intentionally included to be able to assess the program’s suitability to incorporate into a training program, rather than continuing education once employed.

### Conclusions and Future Directions

The Dementia Foundations Program was positively evaluated by a group of 50 unregulated care providers, predominantly PSWs and PSW trainees, with participants showing a significant improvement in both dementia knowledge and attitudes. Participants found the training very useful in supplementing their existing education and training, with particular relevance to the PSW trainee cohort. The findings of this study have implications for unregulated care providers who support people living with dementia, as well as trainees who may not yet interact with those living with dementia. The Dementia Foundations Program is an effective and user-friendly e-learning program that can be conveniently incorporated within existing dementia training and educational programs as part of a blended teaching and learning strategy to enhance dementia knowledge in unregulated care providers and trainees. Widespread implementation has the potential to increase training capacity and fill gaps in existing workforce training and improve dementia knowledge, attitudes, and awareness among care providers as dementia prevalence increases in Canada.

Since this pilot study was conducted, the Dementia Foundations Program has reached 1012 users from December 2021 to September 2024, with approximately 55% completing all 4 courses and receiving a certificate. Postprogram evaluations show high satisfaction with the courses from individuals who purchased the program digitally and employees and students from organizations who purchased the program for professional development.

Future work will focus on the spread and scale of this training program through advertising and partnerships with relevant organizations and institutions, and future research should use implementation science to assess organizational adoption and effectiveness. Continuous quality improvement evaluations will be conducted to determine the best approach for widespread implementation.

## Abbreviations

DAS: Dementia Attitudes Scale
DKAS: Dementia Knowledge Assessment Scale
LTC: long-term care
PSW: personal support worker
UDMOOC: Understanding Dementia Massive Open Online Course
